# Use of Gas Condensate for the Recovery of Hydrocarbons from Oily Sludge

**DOI:** 10.1021/acsomega.6c04519

**Published:** 2026-09-08

**Authors:** André Luis Andrade dos Santos, José Jailton Marques, João Paulo Lobo dos Santos, Gabriel Francisco da Silva, Luiz Carlos Lobato dos Santos, George Simonelli, André Luis Dantas Ramos

**Affiliations:** † 42506 Petrobras, Rio de Janeiro 20231-030, Brazil; ‡ Postgraduate Program in Engineering and Environmental Sciences, 544664 Federal University of Sergipe, Sao Cristovao 49100-000, Brazil; § Postgraduate Program of Chemical Engineering, 74391 Federal University of Sergipe, Sao Cristovao 49100-000, Brazil; ∥ Oil, Gas, and Biofuels Research Laboratory (PGBio), Postgraduate Program in Chemical Engineering (PPEQ), 28111 Federal University of Bahia (UFBA), Salvador 40210-630, Brazil

## Abstract

Petroleum operations generate large volumes of oily sludge, a complex residue composed of emulsified oil, water, mineral solids, and chemical additives. This waste reduces storage capacity, accelerates corrosion, and poses significant environmental challenges. Although gas condensate (C5^+^) has been mentioned in isolated studies, its systematic application as the primary extraction solvent in a mixer–settler system combined with surfactant-assisted demulsification and statistically optimized operating conditions remains unexplored. This study evaluates the feasibility of using C5^+^ to recover hydrocarbons from oily sludge generated during primary processing. A mixer–settler system was employed with C5^+^ as the extraction solvent and the commercial surfactant Dissolvan 974 (Clariant), a polycondensed resin dissolved in an organic solvent mixture of xylene and ethanol (20–30% each), to destabilize emulsions. The raw sludge contained 60.5% water, 23.5% oil, and 16.0% solids. A 3^3–1^ fractional factorial design was used to optimize operational parameters. The results demonstrate that C5^+^ enables efficient oil–water–solid separation and high hydrocarbon recovery, providing a technically viable and environmentally aligned pathway for oily sludge valorization. This integrated strategy offers a cost-effective alternative by utilizing an internal industrial byproduct to replace conventional solvents, while promoting environmental sustainability through circular-economy principles and alignment with the United Nations Sustainable Development Goals (SDGs 6, 9, and 12).

## Graphical abstract

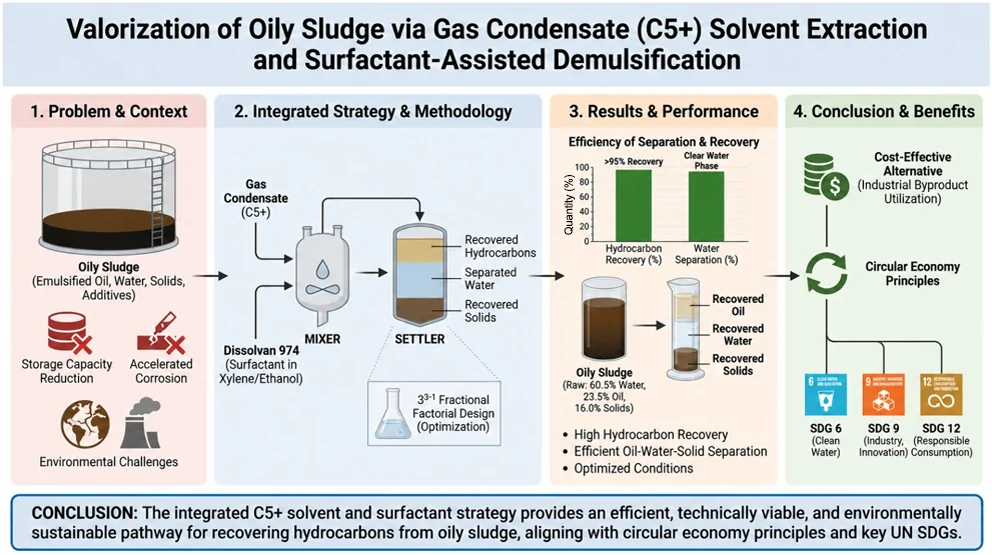

## 1 Introduction

Petroleum exploration, transportation, refining, and storage generate large quantities of oily sludge, making its management a persistent environmental and operational challenge.^1–5^ This residue is a heterogeneous mixture of hydrocarbons, water, mineral solids, heavy metals, and hazardous compounds such as asphaltenes, benzene, and phenols.^1–3^ It also contains inorganic particles from drilling fluids, well-testing discharges, saline wastewater, and formation fines,^6^ forming a complex matrix whose composition varies widely depending on its origin. Typically, oily sludge may contain 15–50% hydrocarbons, 30–85% water, and 5–46% solids.^7,8^

The scale of sludge generation underscores its environmental relevance. Approximately one ton of oily sludge is produced for every 500 tonnes of crude oil processed, contributing to an estimated 160 million tonnes of waste generated annually worldwide.^9,10^ This makes oily sludge one of the largest waste streams in the oil and gas sector, with direct implications for operational reliability and equipment integrity.^6,11^ Its accumulation reduces storage capacity, increases viscosity, and accelerates corrosion in tanks and pipelines, reinforcing the need for treatment technologies capable of handling highly stable emulsions.^6,12^ A wide range of technologies have been developed for hydrocarbon recovery, including mechanical, chemical, biological, and hybrid processes.^5,13^ Common approaches include landfilling, incineration, microwave liquefaction, centrifugation, and solvent extraction.^6^ Treatment efficiency is strongly influenced by sludge composition, viscosity, and the presence of bound water, which stabilizes asphaltene-rich aggregates and hinders separation.^8,14^ Therefore, selecting an appropriate method requires assessing both techno-economic feasibility and life-cycle environmental impacts.^5,15^

Although techniques such as pyrolysis, electrokinetic treatment, and ultrasonic irradiation have shown potential, they are often limited by high capital costs and operational complexity.^5,16^ Process intensification studies have reported oil recoveries of 84–87% using xylene and up to 78.62% with biosurfactants,^17,18^ yet these methods frequently depend on external additives and overlook the use of internal industrial hydrocarbon streams as extraction media. Solvent extraction remains a robust and cost-effective strategy for removing organic compounds, especially when solvent–sludge ratios are properly optimized.^6,14,19^ Compared with traditional methods, extraction-based techniques generally offer lower environmental impacts and higher potential for recovering refinery-grade oil.^15,19^ While hexane and xylenes have achieved recovery rates of 67.5%, and synergistic solvent–surfactant systems up to 97%, the use of natural gas condensate (C5^+^) remains underexplored.^12,20^ This byproduct of natural gas processing, rich in saturated hydrocarbons, exhibits properties similar to petrochemical naphtha.^21^ Reusing C5^+^ as an extraction solvent aligns with circular-economy principles and supports the United Nations Sustainable Development Goals (SDGs 6, 9, and 12).

Despite isolated mentions of gas condensate as an extractant, no previous studies have systematically evaluated C5^+^ as the primary solvent in a mixer–settler system combined with surfactant-assisted demulsification and statistically optimized operating conditions. In this work, C5^+^ is used as an internal NGPU byproduct rather than an external petrochemical solvent, reinforcing industrial circularity and aligning the process with sustainability principles. This integrated approach represents a novel contribution by demonstrating that an internal NGPU byproduct can replace conventional petrochemical solvents while achieving near-complete hydrocarbon recovery. Here, we systematically assess the synergistic interaction between C5^+^ and Dissolvan 974, showing that this combination is more effective at disrupting rigid asphaltene–mineral aggregates than solvent volume alone. In this context, the present study evaluates the use of C5^+^ from a Natural Gas Processing Unit (NGPU) as a solvent for hydrocarbon recovery from oily sludge. We propose a liquid–liquid extraction model combining C5^+^ and surfactants and assess its performance using both centrifugal and gravitational separation routes. This strategy targets highly stable oily sludge with a muscovite-rich mineral matrix, enabling the release of hydrocarbons typically resistant to conventional extraction and achieving recovery levels comparable to refining specifications.^14^

## 2 Methodology

### 2.1 Obtaining and Characterizing Oily Sludge

The oily sludge samples used in this investigation were collected from strategic production units in the Carmópolis field, Sergipe, Brazil. These residues are complex and hazardous semisolid emulsions that contain hydrocarbons, water, and mineral solids.^5,19^ The samples were classified according to their points of generation and accumulation, which directly influence physicochemical properties and colloidal stability.^22^ Four categories were analyzed: AO1 from the API Box at the primary processing station, AO2 from the bottom of the central primary processing tank during maintenance, AO3 from the floated oil supernatant of the produced water flotation unit, and AO4 from the inlet of the secondary hydrocarbon treatment unit. Figure 1 illustrates the potential formation points of these residues.

**Figure 1:**
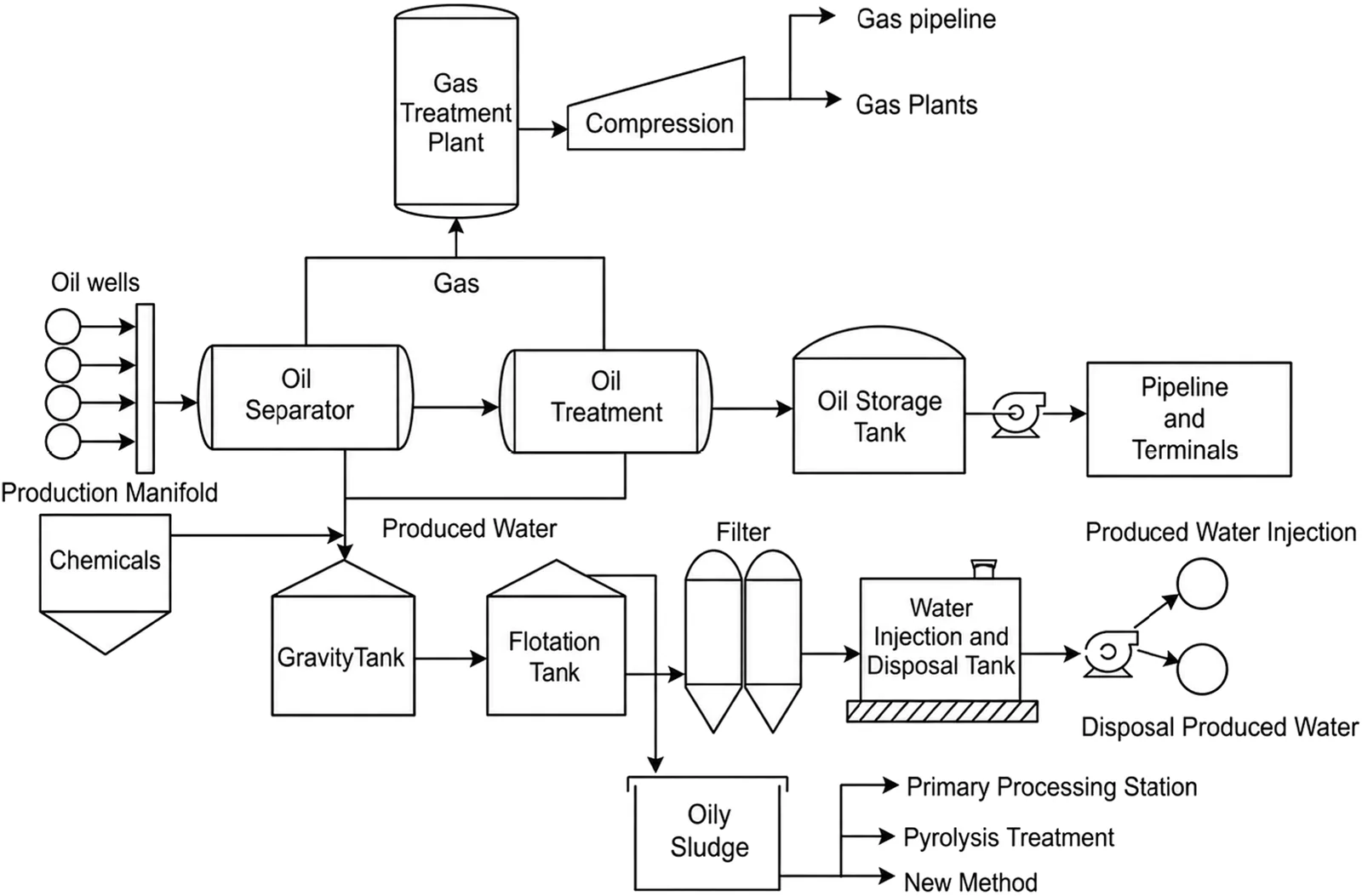
Simplified flowchart of primary processing in oil collecting stations, highlighting the sampling points (AO1, AO2, AO3, and AO4) for oily sludge characterization.

Residues generated from flotation processes, such as AO3, represent a major operational challenge in the region because the central primary processing unit produces approximately 30 m^3^ of oily residue per day. This diversity of samples is essential for evaluating treatment efficiency since sludge properties such as viscosity, density, and sediment accumulation vary substantially with crude oil origin, storage conditions, and the specific processing configuration of each refining unit.^15,22,23^

The oily sludge samples and their constituent fractions were characterized for basic water and sediment content (BSW), total solids, salinity, asphaltenes, and resins according to the standards listed in Table 1. Droplet size distribution was determined by laser diffraction following ISO 13320:2009, which is a key indicator of emulsion stability and droplet coalescence potential.^24,25^ Inorganic solids were analyzed by X-ray diffractometry to identify mineral phases such as quartz and muscovite, which often act as matrices for hazardous hydrocarbon retention.^14,26^ The aqueous phase was characterized by measuring chloride content, salinity, pH, hardness, and density, properties that strongly influence interfacial behavior and the stability of water-in-oil emulsions.^5,27,28^

**Table 1:** Characterization Parameters and Standards Used in the Analysis of the Crude Oily Sludge Sample

parameters	Methods
Basic Water and sediment (BSW)	ASTM 4377-00
Total solids	ASTM D 4807
Salinity	ASTM-6470
Density	ASTM-5002
Droplet size distribution	Difração a Laser ISO – 13320 (2009)
Asphaltene and resin content	ASTM 6560/ASTM-D3921

API gravity was calculated from specific gravity using eq 1.^29^ This index is fundamental for evaluating processing potential and the quality of recovered hydrocarbons.^5,15,30^

1 API°=141,5d−131,5

A preliminary assay was conducted to characterize the inorganic solid fraction. Hydrocarbons were removed through Soxhlet extraction with chloroform at 61.2 °C for 3 h, a step necessary to disrupt asphaltene–solid agglomerates that hinder accurate mineral identification.^14^ The extracted solids were dried at room temperature, milled using a Retsch MM200 ball mill, and sieved through a 325 mesh screen to obtain particles smaller than 0.045 mm. X-ray diffractometry was performed using a Bruker D2 Phaser with Cu Kα radiation at 30 kV and 10 mA, scanning from 5° to 90° with a 0.02° step and 3 s reading time. Mineral phases were identified using the Rietveld Method and compared with the ICDD PDF2 (2011) and the Crystallography Open Database.^26^

### 2.2 Treatment Routes

The experimental prototype used for oily sludge treatment is shown in Figure 2. It consisted of a mixer, a jar test unit (Polilab Turbfloc/2P), and a settling tank with an adapted drain to promote oil–water separation and hydrocarbon recovery. Solvent and surfactant were added to the jar test unit under controlled agitation to ensure intensive mechanical mixing, maximize solute–solvent interaction, and enhance mass transfer within the sludge matrix.^5,12^ This agitation disrupts the rigid interfacial films formed by natural emulsifiers and ensures effective dispersion of chemical agents, facilitating the release of hydrocarbons from cohesive solid–aqueous aggregates.^12,14^

**Figure 2:**
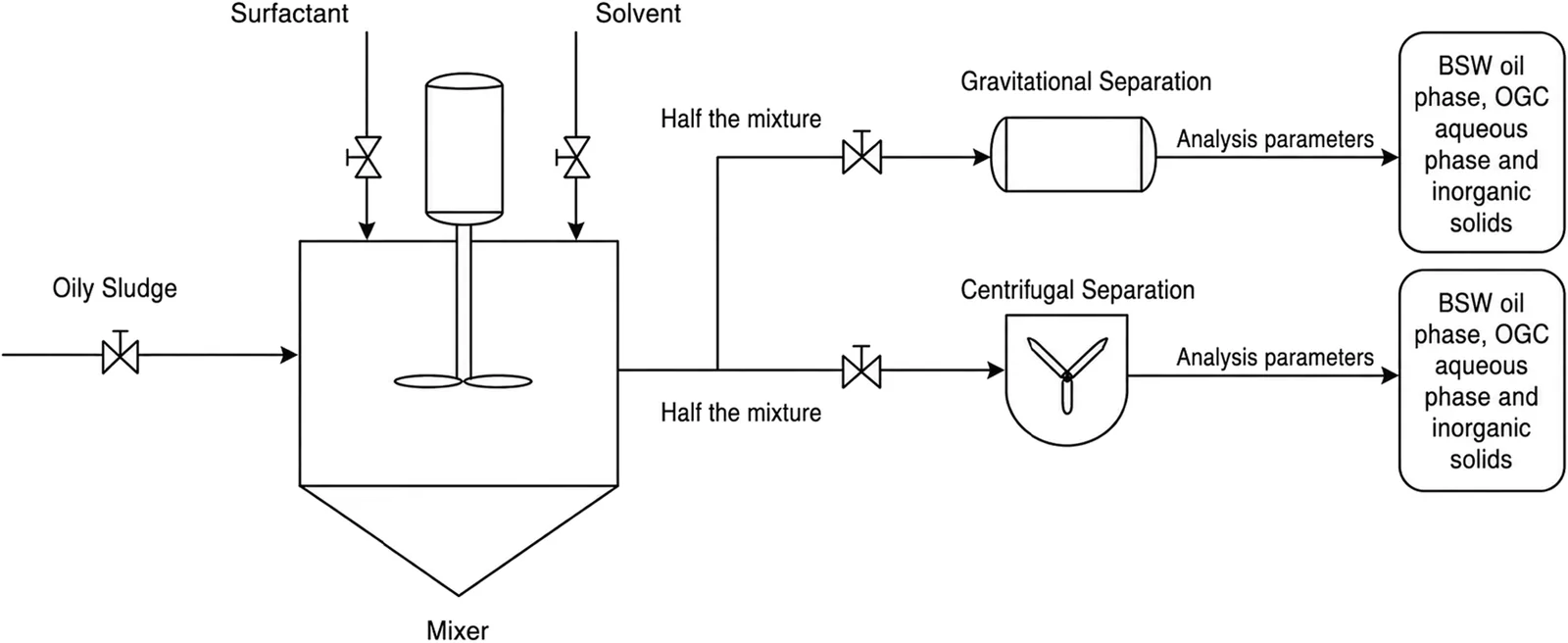
Illustrative diagram of the experimental procedure for the centrifugal and gravitational routes.

Two separation routes were applied. In the gravitational route, samples were transferred to 1000 mL graduated cylinders with an adapted drain, relying on density and size differences between inorganic solids and asphaltene aggregates, as predicted by Stokes’ Law.^14^ In the centrifugal route, samples were processed in an NT 871 Nova Técnica centrifuge operating between 1300 and 1500 rpm, using mechanical forces to overcome the resistance of stable water-in-oil emulsions.^5,31^

### 2.3 Experimental Tests

Operational parameters kept constant included sample mass of 25 g, agitation time of 10 min, mixing speed of 55 rpm, and temperature of 25 °C.^20,32^ To address the heterogeneity of the oily sludge, samples were homogenized by gentle mechanical agitation for 15 min in a sealed container before aliquot extraction, a procedure essential for representative sampling in complex oily waste matrices.^4,22^

The samples underwent a demulsification step to destabilize rigid interfacial films and promote phase separation.^12^ After this stage, specific volumes of natural gas condensate (C5^+^) and the commercial surfactant Dissolvan 974 (Clariant), a polycondensed resin dissolved in an organic solvent mixture of xylene and ethanol (20-30% each), were added to 250 g of oily sludge, following the fractional factorial design detailed in Table 2.^12,21^ This demulsifier is characterized as a violet liquid with a density of approximately 0.9365 g/cm^3^ and a near-neutral pH of between 6 and 7. The mixture was briefly stirred for 20 seconds to ensure uniform dispersion of chemical agents and initiate the release of asphaltenes trapped in water-induced aggregates. The sample was then processed in a jar test unit at 55 rpm for 10 min to enhance mass transfer and maximize solute-solvent interactions.^5,12^ This demulsifying agent was selected for its ability to reduce the viscosity and surface tension through the accumulation of its hydrophilic and hydrophobic groups at the oil-water interface, facilitating the displacement of natural emulsifiers and the subsequent coalescence of water droplets.

**Table 2:** Independent Variables and their Respective Levels^a^

levels		*V*_d_ (mL)	*V*_s_ (mL)	*T*_c_ (min)	*T*_d_ (h)
–1	Low	10	100	5	24
0	Medium	25	200	10	36
1	High	40	300	15	48

a Abbreviations: *V*_d_, surfactant volume; *V*_s_, solvent volume; *T*_c_, centrifugation time; and *T*_d_, settling time.

The C5^+^ solvent used in this study was an industrial natural-gasoline stream obtained from natural gas processing operations in Sergipe, Brazil. The specific batch used in the experiments was not subjected to detailed component-by-component gas chromatographic analysis. Therefore, an exact composition cannot be assigned retrospectively to the experimental solvent. Coelho^33^ characterized an industrial C5^+^ stream from the Petrobras Sergipe–Alagoas natural gas processing context by triplicate gas chromatography. The stream was predominantly paraffinic and contained approximately 27.38 mol % *n*-pentane, 26.80 mol % isopentane, 12.11 mol % *n*-hexane, 9.82 mol % 2,3-dimethylbutane, 5.88 mol % *n*-heptane, 4.00 mol % methylcyclopentane, and 3.92 mol % 3-methylpentane, in addition to smaller amounts of other C6–C12 hydrocarbons. PONA analysis indicated approximately 88.5 mol % paraffins and 11.5 mol % naphthenes. These data are presented only as a representative composition of an industrial C5^+^ stream and not as the analytical composition of the specific batch used herein.^33^

After mixing, the material was transferred to either a graduated cylinder or a centrifuge tube depending on whether gravitational settling or centrifugation was selected. This stage aimed to destabilize the water-in-oil emulsion and use mechanical forces to accelerate the separation of components with different densities.^5,31^ Once the emulsion separated into distinct layers, the oil, aqueous, and solid fractions were fully characterized to assess the treatment performance and resource recovery potential.^4,19^

### 2.4 Experimental Design

To optimize the recovery process and identify the most favorable operating conditions, a fractional factorial design of type 3^3–1^ was applied, consisting of three orthogonal blocks with three factors at three levels. This statistical approach is widely used to assess the individual and combined effects of variables such as solvent volume, surfactant volume, and processing time on separation efficiency.^5,12^ Based on the independent variables and levels shown in Table 2, nine experimental combinations were generated.

The main objective was to maximize hydrocarbon recovery efficiency by evaluating the reduction of basic water and sediment content in the oil phase, a response directly linked to emulsion destabilization and phase separation.^12,32^ Separation efficiency was calculated using eq 2.

2 $$Ef\left(\%\right)=\left(\frac{{BSW}_{i}-{BSW}_{f}}{{BSW}_{i}}\right)\times 100$$

To evaluate the influence of operational factors on hydrocarbon recovery efficiency (*E*_r_), the experimental data were analyzed using multiple linear regression via the least squares method to generate quadratic polynomial models. These models quantitatively describe the individual and interactive effects of surfactant volume (*V*_d_), solvent volume (*V*_s_), and processing time (*t*), identifying synergistic (positive coefficients) or antagonistic (negative coefficients) interactions among the variables. The statistical significance and predictive power of the models were validated through Analysis of Variance (ANOVA), comparing calculated *F*-values against critical *F*-values at a 95% confidence interval, while the quality of fit was assessed by adjusted coefficients. All statistical procedures, including the construction of Pareto charts, response surfaces, and predicted versus observed plots to verify the absence of systematic bias and lack of fit, were performed using Statistica 8.0.

## 3 Result and Discussion

### 3.1 Characterization of Oily Sludge

The physicochemical composition of oily sludge varies considerably because of differences in crude oil origin, refinery processing schemes, and the chemical additives used during production and treatment.^15,22^ This heterogeneity creates operational challenges because properties such as viscosity, density, and sediment accumulation fluctuate with storage conditions and the configuration of each processing unit.^8,23,34^ Detailed characterization is therefore essential for selecting appropriate recovery strategies and ensuring the process efficiency.^5,15^ The main physicochemical properties of the sludge samples collected at different operational points are presented in Table 3.

**Table 3:** Physicochemical Characteristics of Oily Sludge^a^

properties	AO1	AO2	AO3	AO4
Water content (%)	54	65	59	64
Oil content (%)	31	18	29	16
Solid content (%)	15	17	12	20
Salinity (mg/L)	29,125	23,712	31,734	32,347
Specific gravity at 20 °C (g/cm^3^)	0.984	0.994	0.979	0.992
°API	12.37	10.83	12.97	11.14

a AO1, API box of the oil processing station; AO2, bottom of the central oil processing tank (open tank for maintenance); AO3, floated oil (supernatant) from produced water flotation unit; AO4, oily sludge from the inlet of the secondary hydrocarbon recovery treatment unit (SHRTU).

Oily sludge is typically a highly stable water-in-oil emulsion in which water droplets are dispersed within a continuous petroleum phase.^19,35^ The samples analyzed in this study exhibited water contents of 54 to 65 percent and solid fractions of 12 to 20 percent, values consistent with those expected for hazardous oily wastes generated during primary processing and storage operations.^5,15,22^ This agreement with literature data confirms that the sludge from the Carmópolis field displays the characteristic stability and heterogeneity of industrial petroleum residues.^5,19,22^

Inorganic solids present in oily sludge act as natural surfactants that reinforce emulsion stability by forming rigid interfacial films.^36,37^ These particles accumulate at the water–oil interface and generate Pickering emulsions in which solid-stabilized barriers prevent droplet coalescence.^5,38^ Recent studies indicate that mineral phases associated with bound water promote the formation of asphaltene–solid agglomerates that are highly resistant to conventional separation.^5,14^ This entrapment mechanism alters adsorption and desorption equilibria at solid–liquid interfaces.^5,39^ These effects explain why samples AO4, AO2, and AO1 exhibit greater stability than AO3. Their higher solid and resin contents favor the formation of more rigid viscoelastic films, reducing interfacial tension, and increase the treatment intensity required for effective hydrocarbon recovery.^5,15^

Salinity is another critical factor influencing emulsion stability because higher salt concentrations generally increase resistance to separation regardless of oil type.^40^ Elevated salinity also poses operational risks due to its corrosive potential and its tendency to promote scaling in pipelines and industrial equipment.^27,28^ Emulsions with high basic sediment and water content typically become more stable in the presence of sodium, calcium, magnesium, and potassium salts.^41^ In this study, salinity values ranged from 23,712 to 32,347 mg/L, which are moderate compared with crude oils that may contain up to 200,000 mg/L of total salts.^42^ According to the classification of Pabón and Souza Filho,^43^ oils with API gravity between 10.0 and 22.3 degrees are considered heavy. All sludge samples fell within this range, with values between 10.83- and 12.37-degrees API, confirming the heavy and complex nature of the Carmópolis residues.^5,15^

### 3.2 Characterization of the Oil Phase

Oily sludge is a complex mixture composed of saturated and aromatic hydrocarbons, resins, asphaltenes, and oxygenated compounds such as ketones and alcohols.^44^ Among these components, resins and asphaltenes are particularly relevant because they act as natural emulsifiers that form rigid and viscous interfacial films capable of stabilizing water in oil emulsions and inhibiting droplet coalescence.^15,24,30^ In this study, the oily phase was characterized in terms of droplet size distribution and resin and asphaltene contents, as shown in Table 4. Droplet size is fundamental for assessing colloidal stability and predicting phase separation, while quantifying heavy fractions provides insight into the formation of asphaltene–solid aggregates that hinder conventional recovery.^24,25^ These components promote hydrocarbon entrapment within mineral matrices and generate complex aggregates that require specialized treatment for effective extraction.^5,14^

**Table 4:** Asphaltene and Resin Content in the Oily Phase of Petroleum Oily Sludge and Distribution of their Respective Droplet Sizes in the Synthesized Emulsion

properties	AO1	AO2	AO3	AO4
Asphaltenes (%)	5.80	7.40	6.80	9.10
Resins (%)	2.80	4.30	3.40	5.30
D [4,3]	79.0	48.0	56.5	39.0
D [0,5]	71.8	40.8	48.2	32.6

Heavy oils typically contain elevated levels of heteroatoms and substantial amounts of resins and asphaltenes.^30^ These fractions strongly influence emulsion stability due to their interfacial activity, acting as natural emulsifiers within the sludge matrix.^5,37,45^ Asphaltenes and resins adsorb onto water droplet surfaces and form interfacial films that inhibit coalescence and hinder phase separation.^46,47^ Their stabilization effect arises from complex molecular structures and synergistic behavior. Asphaltenes form irreversibly adsorbed viscoelastic films, while resins reduce interfacial tension through solvation at the interface.^24,48^ Recent studies also show that these heavy fractions promote the formation of asphaltene–solid agglomerates in the presence of bound water, generating toluene-unextractable organic material that requires specialized chemical intervention for recovery.^5,14^

Droplet size distribution is equally important, as droplet diameter directly influences particle packing and emulsion stability.^24^ Emulsions composed of smaller droplets exhibit higher colloidal stability and are less susceptible to coalescence or phase separation.^24^ Traditional separation methods are generally ineffective for droplets smaller than 20 μm, which remain suspended in the continuous phase.^25,49^ This stability is reinforced by the presence of resins and asphaltenes, the main components governing interfacial rheology in heavy oil systems.^5,30^ Asphaltenes form rigid viscoelastic films that act as mechanical barriers to droplet growth, while resins form less dense solvated films that reduce interfacial tension and act synergistically with asphaltenes.^24,46,48^ These heavy polar fractions also promote the formation of asphaltene–solid agglomerates in the presence of bound water, trapping hydrocarbons within mineral matrices.^5,14^

Laser diffraction analysis provides key parameters such as the median diameter D[0.5] and the volume mean diameter D[4.3].^50^ D[0.5] represents the diameter below which 50 percent of the particle volume is found, while D[4.3] corresponds to the diameter of a sphere with the same average volume as the particles in suspension.

### 3.3 Characterization of the Water Phase

On the basis of the characterization of the oily phase, sample AO3 was selected for further investigation because it exhibited lower solids content, reduced resin and asphaltene levels, and a smaller droplet size distribution compared with sample AO1. This selection reduced the total number of experiments while maintaining a representative sludge matrix with specific colloidal characteristics. The physicochemical analysis of the produced water associated with AO3 revealed a salinity of 32 643.1 mg/L, total hardness of 10 195.10 mg/L, and chloride content of 56 147.0 mg/L. The aqueous phase also contained 15.7 mg/L suspended solids and had a pH of 6.7. These high salinity levels are technically relevant because they enhance emulsion stability and influence interfacial behavior during oil processing.^51,52^ The pH of 6.7 further contributes to stability because particle-to-particle repulsion decreases below pH 7.0 due to the protonation of carboxylic groups.^53^ The smaller droplet size distribution observed in AO3 is also associated with greater colloidal stability, indicating that such emulsions are less prone to coalescence and are more resistant to phase separation.^24^

High salinity is a critical parameter governing emulsion behavior because saline environments increase interfacial elasticity and promote the formation of more rigid films at the oil–water interface.^51^ Studies show that while average droplet diameters tend to increase in non-saline media, they remain unchanged under high salinity, which indicates greater resistance to coalescence.^40^ Specific ions such as sulfates and chlorides further reinforce stability by forming compact interfacial layers that modify electrostatic interactions and lead to smaller droplet sizes.^54^ Crude oils with high basic water and sediment content also exhibit increased stability in the presence of sodium, calcium, magnesium, and potassium salts.^41^ In industrial operations, these saline compounds not only hinder phase separation but also accelerate corrosion and promote scale formation in pipelines and processing equipment.^27,28^

The pH of the medium also plays a decisive role in particle interactions. Below pH 7.0, protonation of carboxylic groups reduces electrostatic repulsion, which increases emulsion stability and resistance to coalescence.^52,53^ Recent studies also indicate that saline aqueous phases can induce the formation of asphaltene–solid aggregates in the presence of bound water, generating unextractable organic fractions that require specialized treatment.^14^ Together, these physicochemical characteristics, including high salinity, near-neutral pH, and the smaller droplet size distribution of AO3, highlight the strong colloidal stability of the system and justify its selection for subsequent analyses.

### 3.4 Characterization of the Solid Phase

The main inorganic solids identified in the oily sludge by XRD are listed in Table 5. These minerals include calcite, kaolinite, halite, pyrite, quartz, muscovite, strontian barite, siderite, goethite, and albite, with muscovite and pyrite representing the most abundant phases.

**Table 5:** Main Inorganic Solids Found in Petroleum Oily Sludge by XRD

mineral	fórmula	mean (%)
Calcita	CaCO_3_	9.46
Caulinita	Al_2_Si_2_O_5_(OH)_4_	13.42
Halita	NaCl	3.47
Pirita	FeS_2_	20.72
Quartzo	SiO_2_	9.57
Muscovita	KAl_2_(Si_3_Al)O_10_(OH,F)_2_	24.90
Barita estronciada	Ba_0,75_Sr_0,25_SO_4_	9.75
Siderita	FeCO_3_	3.10
Goethita	FeO(OH)	3.21
Albita	NaAlSi_3_O_8_	2.39

The separation of inorganic solids is a fundamental requirement for effective hydrocarbon recovery from complex sludge matrices.^36^ In heavy oil production environments such as the Liaohe oilfield, multi-stage chemical treatments are often required to isolate these mineral phases.^36^ Inorganic residues may also contain naturally occurring radioactive materials, which pose operational and environmental risks that demand costly management strategies.^55^ In the Brazilian oil industry, radioactivity has been correlated with chemical composition, particularly in residues dominated by strontium barite.^56^ This mineral fraction, along with calcium carbonate and silicates, promotes the formation of asphaltene–solid agglomerates in the presence of bound water.^14^ These agglomerates trap hydrocarbons within the solid matrix and generate a toluene-unextractable organic fraction that hinders phase separation and reduces recovery yields.^14^ Therefore, comprehensive characterization of these inorganic components is essential for optimizing treatment strategies and ensuring the sustainable valorization of petroleum waste streams.^5,14^

In this study, muscovite was identified as the predominant mineral phase, with an average concentration of approximately 25 percent, as shown in Table 5. This reflects the geological characteristics of the Carmópolis field, where this silicate is abundant. Muscovite is commonly reported in oil-producing regions and is not typically associated with elevated radioactivity levels, which is advantageous for waste handling and disposal.^28,57^ Unlike strontium barite, which is frequently correlated with radioactive isotopes in Brazilian petroleum residues, the muscovite-rich sludge analyzed here likely avoids the strict management protocols required for naturally occurring radioactive materials.^55,56^ Nevertheless, these inorganic particles can still promote the formation of asphaltene–solid agglomerates in the presence of bound water.^14^ These agglomerates trap hydrocarbons within the mineral matrix and generate organic fractions unextractable by conventional methods, thereby reducing recovery efficiency.^5,14^ Thus, detailed characterization of these solids is essential for optimizing treatment strategies and enabling the sustainable valorization of petroleum waste streams.^5,15^

The mineralogical results obtained in this study align with findings from other production regions such as Taiwan, where drilling cuttings contain barite, quartz, muscovite, calcite, kaolinite, and chlorides.^57^ The reuse of oily wastes in ceramic materials or road pavement bases is considered an economically and environmentally viable alternative for managing petroleum byproducts.^9,57^ While Al Attar et al.^55^ reported strontium barite and hokutolite as dominant phases in specific industrial scales, studies from the Potiguar Basin and the Presidente Bernardes Refinery identified quartz, calcite, kaolinite, and muscovite as the main mineral constituents.^28,58^ Minerals such as quartz are particularly relevant because they promote the formation of asphaltene–solid agglomerates in the presence of bound water, trapping hydrocarbons and generating organic fractions resistant to conventional extraction.^5,14^ Therefore, detailed mineralogical characterization is essential for optimizing treatment strategies and improving hydrocarbon recovery from petroleum waste streams.^5,15^

### 3.5 Efficiency of the Mixer–Settler System in Phase Separation

In solvent extraction, oily sludge is mixed with organic solvents in controlled proportions to facilitate hydrocarbon removal.^19,59^ Several solvents have been reported in the literature, including hexane,^60^ dichloromethane,^61^ and binary mixtures such as acetone and hexane in a 1:1 ratio.^62^ Other systems employ combinations of cycloalkanes and *n*-alkanes.^63^ The oily residues examined in this study originate from petroleum production in the Carmópolis Field of the Sergipe–Alagoas Basin, whose crude oils have been previously characterized. These characterizations revealed notable variability among the field’s crude oil blends, particularly in terms of their physicochemical properties and the behavior of their heavy fractions.^64^ The superior extraction performance obtained with C5^+^ can be attributed to its predominantly nonpolar composition, low-molecular weight, strong dilution capacity, and multicomponent nature. Light hydrocarbon solvents are known to mobilize and extract oil from petroleum sludges.^20,22^ Unlike a pure solvent, industrial C5^+^ contains normal and branched paraffins and cycloalkanes mainly within the C5–C7 range, along with smaller amounts of heavier hydrocarbons.^33^ This compositional diversity likely broadens the effective solvency window and promotes a co-solvency effect, enabling different constituents of the mixture to interact with distinct portions of the recoverable petroleum fraction. The reduction in oil viscosity produced by low-viscosity C5^+^ hydrocarbons further enhances solvent penetration, mass transfer, and detachment of oil from solid particles. The observed extraction efficiency is therefore most plausibly associated with dissolution and mobilization of the maltene-rich fraction rather than complete solubilization of the residue, since low-molecular-weight paraffinic hydrocarbons have limited solvency toward asphaltenes and may induce their precipitation.^65^ Because SARA fractionation was not performed before and after extraction, this mechanism should be interpreted as a literature-supported explanation of the experimental results. In particular, previous characterizations of Carmópolis crude oils have shown that certain blends, such as CEOL3, contain heavier and more polar fractions with stronger affinity for produced water, which supports the interpretation adopted here in the absence of direct compositional verification.^64^

High recovery efficiencies reported for these solvents are generally attributed to their ability to reduce oil viscosity and weaken interactions among heavy components such as asphaltenes and resins.^7,66,67^ Although solvent extraction is considered a simple and rapid method for obtaining high-quality hydrocarbons, it still faces limitations related to high solvent consumption and potential secondary environmental impacts.^59,68^ The precipitation of heavy fractions further promotes phase separation within the sludge matrix.^5,7,66^ Since water and solids are immiscible with organic solvents, their removal typically requires additional mechanical steps such as centrifugation or gravitational settling.^19,59^ In this study, however, the organic phase was recovered using a mixer–settler system with natural gas condensate (C5^+^) and surfactants, eliminating the need for these separate operations.^59,68^ The method relies on the strong solvency of C5^+^ to dilute heavy oil, while the surfactant destabilizes interfacial films that hinder droplet coalescence.^5,12^ Table 6 presents the BSW values and hydrocarbon recovery efficiencies obtained after extraction with C5+ and surfactant for both centrifugal and gravitational separation processes across the nine experiments conducted according to the experimental design.

**Table 6:** Results of Centrifugal and Gravitational Separation as Predicted in the Experimental Design^a^

	independent variables			centrifugal separation (CS)		gravitational separation (GS)	
tests	*V*_d_	*V*_s_	*t*	BSW (%)	*E*_r_ (%)	BSW (%)	*E*_r_ (%)
1	–1	1	0	0.70	98.80	10.00	83.30
2	0	0	0	11.00	81.65	34.00	43.30
3	0	–1	1	20.00	66.70	26.00	56.70
4	1	1	1	17.00	71.70	34.00	43.30
5	0	1	–1	1.00	98.30	30.00	50.00
6	1	–1	0	27.00	55.00	38.00	36.70
7	–1	–1	–1	7.00	88.30	36.00	40.00
8	1	0	–1	10.00	83.30	40.00	33.30
9	–1	0	1	7.00	83.30	38.00	36.70

a *V*_d_, surfactant volume; *V*_s_, solvent volume; *T*_c_, centrifugation time; BSW, free water and sediment content; and *E*_r_, hydrocarbon recovery efficiency.

Maximum oil recovery efficiencies of 99.8 percent were achieved using the centrifugal process and 83.30 percent using the gravitational process, as shown in Table 6. The centrifugal route outperformed values reported in the literature, where recoveries of 94 to 95 percent were obtained with solvents such as naphtha, *n*-heptane, toluene, dichloromethane, dichloroethylene, and diethyl ether, and up to 97 percent with *n*-heptane and kerosene.^68^ Its superior performance is attributed to the intense mechanical forces that rapidly separate components of different densities.^31,59^ Recent studies indicate that recovery rates above 98 percent are characteristic of advanced systems that combine specialized solvents with surfactants to break complex water in oil emulsions by reducing viscosity and destabilizing interfacial films that trap hydrocarbons within the solid–liquid matrix.^5,12,14,66^ Thus, the mixer–settler system integrated with C5+ represents a highly effective alternative for the remediation and valorization of hazardous oily sludge.^12,15^

The hydrocarbon recovery efficiencies obtained in this investigation were technically significant for both processing routes, especially centrifugal separation, reinforcing the industrial feasibility of the methodology. Because a high initial surfactant dosage was used, additional tests with reduced concentrations were conducted to optimize the gravitational process and evaluate more economical operating conditions, as shown in Table 7. This optimization is vital because excessive surfactants can hinder phase separation by forming nanodemulsifying films that inhibit droplet coalescence.^45^ Under these optimized conditions, maximum oil recovery efficiencies of 99.9 percent for the centrifugal route and 99.8 percent for the gravitational route were achieved, resulting from the synergistic interaction between the natural gas condensate and the surfactant.

**Table 7:** Results of Additional Tests for Centrifugal and Gravitational Separation^a^

	independent variables			centrifugal separation (CS)		gravitational separation (GS)	
tests	*V*_d_	*V*_s_	*T*	BSW (%)	*E*_r_ (%)	BSW (%)	*E*_r_ (%)
10	–1.33	1	1	0.05	99.99	0.90	98.50
11	–1.53	0	1	3.00	95.00	23.00	61.70
12	–1.33	0	0	1.90	96.80	10.00	83.30
13	–1.20	0	0	1.00	98.30	0.10	99.80

a BSW, basic water and sediment content; *E*_r_, hydrocarbon recovery efficiency.

When compared to literature benchmarks, the performance obtained in this investigation is markedly superior. Hamidi et al.^70^ reported a maximum of 67.5 percent with hexane and xylene, while Zhao et al. (2019)^71^ found recovery efficiencies of 94 percent, 75 percent, 72 percent, and 81 percent for ethylbenzene, cyclohexane, *n*-heptane, and decalin, respectively. Even the best recoveries typically reported, which range from 80 percent to 97 percent, remain below the efficiencies achieved in this study.

Although the solvent volume was optimized at 300 mL in this investigation, higher hydrocarbon recovery efficiencies were still achieved. This contrasts with the findings of Zubaidy and Abouelnasr,^32^ who reported increasing oil recovery with increasing solvent volume. Similar discrepancies were observed when compared with Guimarães et al.,^58^ who obtained maximum recoveries using 400 mL of hexane and volumes above 300 mL of C5+, respectively. This behavior is scientifically explained by the existence of a threshold for solvent addition, as highlighted by Farrokhi et al.,^45^ who demonstrated that exceeding a critical solvent volume can reduce recovery efficiency. In this study, the high performance achieved with a lower volume of natural gas condensate is attributed to the reduction of oil viscosity and the destabilization of rigid interfacial films promoted by the synergistic interaction between the solvent and the surfactant. Consequently, the ability to break and disperse solid aggregates using optimized chemical dosages emerges as a more decisive factor for near-complete hydrocarbon recovery than simply increasing the solvent-to-sludge ratio.

Centrifugation showed a direct positive relationship with the response variable, resulting in a 25 percent increase in the phase separation efficiency. This technology offers several advantages, including operational simplicity, adaptability, compact equipment design, and high oil recovery capacity without generating secondary pollution.^31,59^ High rotational speeds generate strong centrifugal forces that rapidly separate components of different densities, such as water, solids, oil, and emulsions, while longer centrifugation times further enhance emulsion sedimentation, system destabilization, and oil removal efficiency.^72^ The response variable improves with an increasing speed up to 4000 rpm, a range that effectively promotes droplet coalescence.^72^ However, excessive surfactant concentrations may reverse this effect because the formation of nanodemulsifying films around water droplets inhibits coalescence and reduces the separation efficiency.^45^

Surfactant treatment is based on the principle of chemical cleaning, in which the hydrophilic group of the surfactant interacts with the aqueous phase to improve the solubility of petroleum hydrocarbons.^59^ During oil recovery, the hydrophobic groups accumulate at the oil–water interface, reducing viscosity and surface tension while enhancing the migration of hydrocarbons between phases.^59,73^ This mechanism promotes oil–water demulsification and effective solid–liquid separation, similar to the improvements in rheological performance observed when using waste-derived additives to modify wax crystal structures in crude oils.^59,73^ The addition of chemical agents such as demulsifiers and flocculants further alters the physical and chemical properties of colloidal particles, modifying sludge stability and creating clearer boundaries between the oil phase and the solid–liquid phases.^31,74^ These changes allow rapid separation when combined with high-speed centrifugation.^31,74^ This chemical step can also be integrated with other industrial processes, including electrical methods and flotation.^59^ Compared with alternative technologies that require complex and costly equipment, surfactant treatment is technically simple to implement and does not depend on large mechanical installation.^19,59^

Figure 3 presents the results of the BSW tests after the extraction process, followed by solvent addition and surfactant addition. These results illustrate the progressive reduction in water and sediment contents as chemical and mechanical treatments are applied.

**Figure 3:**
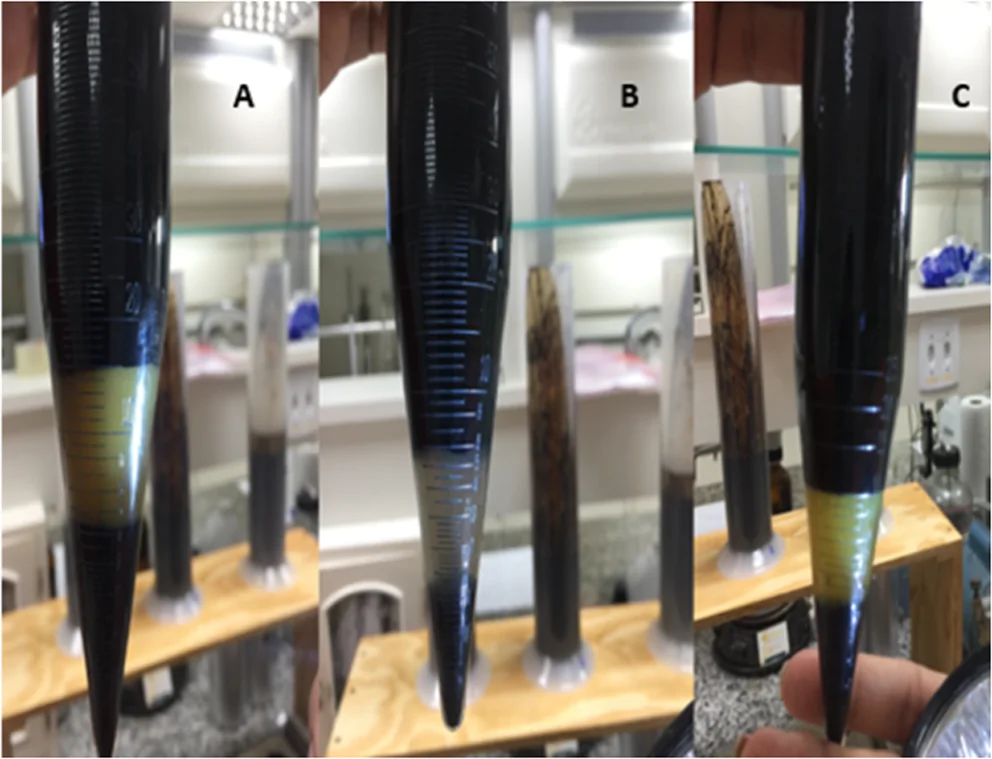
Visual results of BSW tests showing the progressive phase separation: (A) raw sludge after extraction, (B) effect of C5^+^ solvent addition, and (C) complete demulsification after surfactant addition.

Determining appropriate demulsifier dosage rates is inherently complex because of the wide variety of chemical products and the diverse types of crude oil encountered in industrial operations.^74^ The required concentration is highly site-specific and depends on environmental and operational conditions.^75^ Although some studies recommend low dosages between 10 and 50 ppm for effective phase separation, tertiary oil recovery processes may require concentrations in the thousands of ppm.^7,75^ This wide variation occurs because a higher emulsifier content in the sludge demands proportionally higher demulsifier dosages to achieve successful treatment.^75^

In this investigation, additional experiments were conducted to evaluate system behavior at concentrations even lower than those typically recommended in the literature, as shown in Table 7. Surfactant treatment operates through the interaction of hydrophilic and hydrophobic groups that accumulate at the oil–water interface, reducing viscosity and surface tension in the oily sludge.^59^ This mechanism destabilizes rigid interfacial films, promotes droplet coalescence, and enhances hydrocarbon migration between phases.^5,12^ Ultimately, comprehensive sludge characterization and careful optimization of chemical dosages are essential for maximizing recovery efficiency and ensuring the sustainable management of petroleum waste streams.^5,14^

The demulsification or centrifugation time also showed a positive influence on the phase separation in this investigation.^69^ This trend is consistent with that of Zhao et al. (2019),^70^ who reported that extending extraction time from 0 to 10 min increased oil recovery from 10 percent to 70 percent using ethylbenzene, reaching 87.9 percent after 40 min. Studies employing digital baffle batch extraction units similarly indicate that increasing the extraction time from 10 to 20 min enhances fuel recovery by increasing molecular collisions and the interaction between oil droplets and chemical agents. Centrifugation duration is equally important because longer processing times improve emulsion sedimentation, destabilize the sludge structure, and strengthen the separation of components with different densities when they are combined with optimized rotational speeds. The need for intensive treatments to overcome the stability of water in heavy oil emulsions is well-established, since natural surfactants such as asphaltenes and resins form rigid interfacial films that inhibit coalescence. Combined strategies involving gravity separation, heating, centrifugation, hydrocyclones, and chemical additives remain widely applied.^67^ Advanced hybrid systems, including ultrasonic pretreatment coupled with centrifugation, have been shown to increase recovery yields by 15 to 20 percent while reducing the processing time. Overall, optimizing contact time and mechanical forces is fundamental for releasing hydrocarbons trapped within solid and water aggregates and for achieving high-quality resource recovery from petroleum waste streams.

### 3.6 Statistical Analysis on the Effect of Independent Variables on the Response Variable

Regression models were developed for both the centrifugal and gravitational routes. For each route, linear and quadratic equations were evaluated to identify polynomials with high *F* values, low *p* values, and high coefficients of determination. Equations 3,4 represent the regression models applied to the recovery efficiencies for the centrifugal and gravitational processes, respectively. Positive coefficients indicate synergistic effects of the independent variables on recovery efficiency, while negative coefficients indicate antagonistic effects.

3 $$E_{c}=80.78-10.07\times V_{d}+1.08\times {V_{d}}^{2}+9.80\times V_{s}+1.48\times {V_{s}}^{2}-8.03\times t-1.73\times t^{2}$$

4 $$E_{g}=47.03-7.78\times V_{d}+2.23\times {V_{d}}^{2}+7.20\times V_{s}-6.95\times {V_{s}}^{2}+2.23\times t+5.55\times t^{2}$$

where *E*_c_ is the recovery efficiency for the centrifugal route, *E*_g_ is the recovery efficiency for the gravitational route, *V*_d_ is the coded surfactant volume, *V*_s_ is the coded solvent volume, and *t* is the coded time.

Table 8 presents the results of the analysis of variance (ANOVA) for the models. This analysis is crucial for evaluating the statistical significance of the regression equations that were obtained.

**Table 8:** ANOVA for Centrifugal Separation (CS) and Gravitational Separation (GS)^a^

	SS		Df		MS		*F*		*P*-value	
Factor	SC	SG	SC	SG	SC	SG	SC	SG	SC	SG
Vd L+Q	617.272	403.087	2	2	308.636	201.543	24.640	0.775	0.039	0.563
Vs L+ Q	593.645	697.460	2	2	296.822	348.730	23.967	1.341	0.040	0.427
t L+Q	411.012	276.347	2	2	205.506	138.173	16.406	0.531	0.057	0.653
Error	25.052	520.127	2	2	12.526	260.063				
Total SS	1646.980	1897.020	8	8						

a SS, sum of squares; Df, degrees of freedom; MS, mean square; *F*, *F* test value. For CS: calculated F: 21.58; critical F: 19.33; R2:0.98479; adjusted R2:0.93916. For GS: calculated F: 0.88; critical F: 19.33; R2:0.72582; adjusted R2:0.72582.

The polynomial regression model developed for the centrifugal route showed statistical significance because the calculated *F*-value of 21.58 exceeded the critical *F*-value of 19.33. The adjusted coefficient of determination indicated that 93.92 percent of the variability between experimental and predicted recovery efficiencies was explained by the model, while the coefficient of determination of 98.48 percent confirmed a strong agreement between observed and calculated values. The proximity of these coefficients demonstrates that all statistically relevant terms were retained. In contrast, the polynomial model for the gravitational route was not statistically significant because its calculated *F* value of 0.88 was lower than the critical value of 19.33. This indicates that the model cannot be used for the process interpretation. Such statistical validation through ANOVA is essential for optimizing the operational parameters in liquid extraction systems within the petroleum industry.

The Pareto chart for the centrifugal route (Figure 4a) shows that all three independent variables are statistically significant in their linear forms. For the gravitational route, no variable is statistically significant either linearly or quadratically, as shown in Figure 4b.

**Figure 4:**
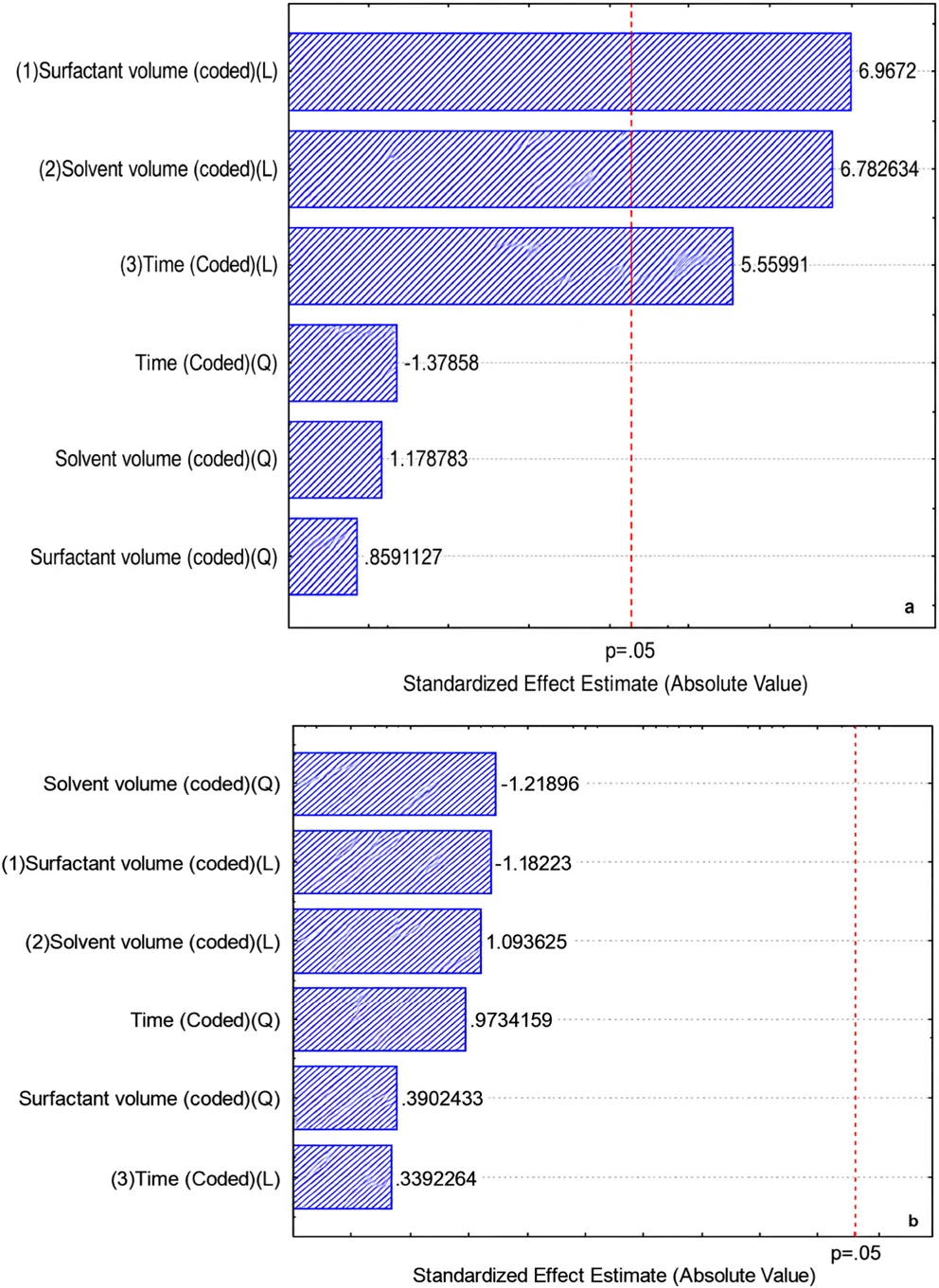
(a) Pareto charts for centrifugal separation and (b) gravitational separation.

To understand the isolated effect of each independent variable, the factor analysis results presented in Tables 9 and 10 reveal clear trends. In the centrifugal process, increasing the treatment time and surfactant volume individually reduced the hydrocarbon recovery efficiency. Centrifugation time caused a 16.07 percent decrease, while the surfactant volume caused a 20.13 percent decrease. This antagonistic behavior is attributed to excessive surfactant concentrations forming nanodemulsifying films around water droplets, which inhibit coalescence and hinder phase separation.^45^ In contrast, increasing the volume of natural gas condensate from 100 to 300 mL improved the recovery efficiency by 19.60 percent.

**Table 9:** Analysis of Factors for Centrifugal Separation

factor	effect	Std Err	*t*(2)	*p*	–95% Cnf.Limt	+95% Cnf.Limt
Mean/Interc.	80.78	1.180	68.47	0.0002	75.71	85.86
*V*_d_ (L)	–20.13	2.890	–6.967	0.0200	-32.57	–7.700
*V*_d_ (Q)	2.150	2.502	0.859	0.4810	-8.618	12.92
*V*_s_ (L)	19.60	2.890	6.782	0.0210	7.166	32.03
*V*_s_ (Q)	2.950	2.502	1.179	0.3600	–7.818	13.72
*t* (L)	–16.07	2.890	–5.560	0.0310	–28.50	–3.633
*t* (Q)	–3.450	2.502	–1.378	0.0300	–14.22	7.318

**Table 10:** Analysis of Factors for Gravitational Separation

factor	effect	Std Err	*t* (2)	*p*	–95% Cnf.Limt	+95% Cnf.Limt
Mean/Interc.	47.03	5.375	8.749	0.013	23.90	70.16
*V*_d_ (L)	–15.57	13.17	–1.182	0.359	–72.22	41.09
*V*_d_ (Q)	4.450	11.40	0.390	0.734	–44.61	53.51
*V*_s_ (L)	14.40	13.17	1.094	0.388	–42.25	71.05
*V*_s_ (Q)	–13.90	11.40	–1.219	0.347	–62.96	35.16
*t* (L)	4.467	13.17	0.339	0.767	–52.19	61.12
*t* (Q)	11.10	11.40	0.973	0.433	–37.96	60.16

For the gravitational route, extending settling time from 24 to 48 h increased recovery efficiency quadratically by 11.1 percent, while increasing solvent volume from 100 to 300 mL produced a linear improvement of 14.40 percent. This positive correlation reflects the ability of natural gas condensate to reduce oil viscosity and disperse solid aggregates, thereby releasing hydrocarbons trapped within the mineral–water matrix.^14^ Based on these findings, the optimal conditions identified in this study include the maximum solvent volume of 300 mL, minimum centrifugation time of 5 minutes, and minimum surfactant volume of 10 mL. Managing phase separation duration remains essential because sedimentation time directly influences extraction efficiency and improves the overall accuracy and precision of the methodology.^72^

During the emulsion separation process, the coalescence of the water droplets was clearly observed. This behavior was facilitated by the surfactant, whose hydrophilic and hydrophobic groups act at the droplet interface to improve solubility and reduce interfacial tension.^59,76^ The surfactant initially displaces natural emulsifiers such as asphaltenes and resins that form rigid interfacial films, preventing coalescence.^48^ This destabilization allows droplets to collide and agglomerate into larger structures.^12^ These enlarged droplets subsequently settle at the bottom of the system, enabling the efficient separation of the water and oil phases through gravitational segregation.^77^

The excellent predictive capability of the centrifugal model is attributed to the application of external mechanical energy, which overcomes the structural stability of the heavy oil emulsion and forces the coalescence of droplets smaller than 20 μm that would otherwise remain suspended.^5,8,13–15^ Centrifugation aligns the response variable more predictably with the experimental factors because high rotational speeds and longer processing times effectively promote droplet sedimentation and system destabilization.^41–43^ Conversely, the statistical insignificance of the gravitational model arises from the high colloidal stability of the sludge matrix. The presence of muscovite and other inorganic solids creates a Pickering effect and rigid viscoelastic films that significantly resist Stokes’ Law-based settling under ambient conditions.^36–38,46,48^ In such complex systems, gravitational separation becomes stochastic and highly sensitive to internal interactions, such as high salinity increasing interfacial elasticity and promoting scaling and corrosion risks.^51–53^ This complexity prevents the quadratic polynomial from establishing a reliable correlation, highlighting the necessity of advanced predictive modeling and statistical optimization to resolve nonlinear interactions in petroleum waste management. Beyond technical recovery rates, the transition to industrial-scale operations necessitates a robust strategy for solvent recycling to ensure economic and environmental sustainability. Although this study focused on maximizing hydrocarbon extraction, the systematic reuse of C5^+^ through distillation-based recovery is a fundamental requirement for industrial implementation to minimize operational costs and maintain the process’s circularity.

### 3.7 Response Surface Analysis

Response surface and contour plots were generated only for the centrifugal route because it was the statistically significant model. These plots, shown in Figure 5a,b, allow the evaluation of interactions between independent variables and their effects on recovery efficiency. Since the model contains three independent variables, each plot was constructed using two variables while keeping the third fixed at its central point value.

**Figure 5:**
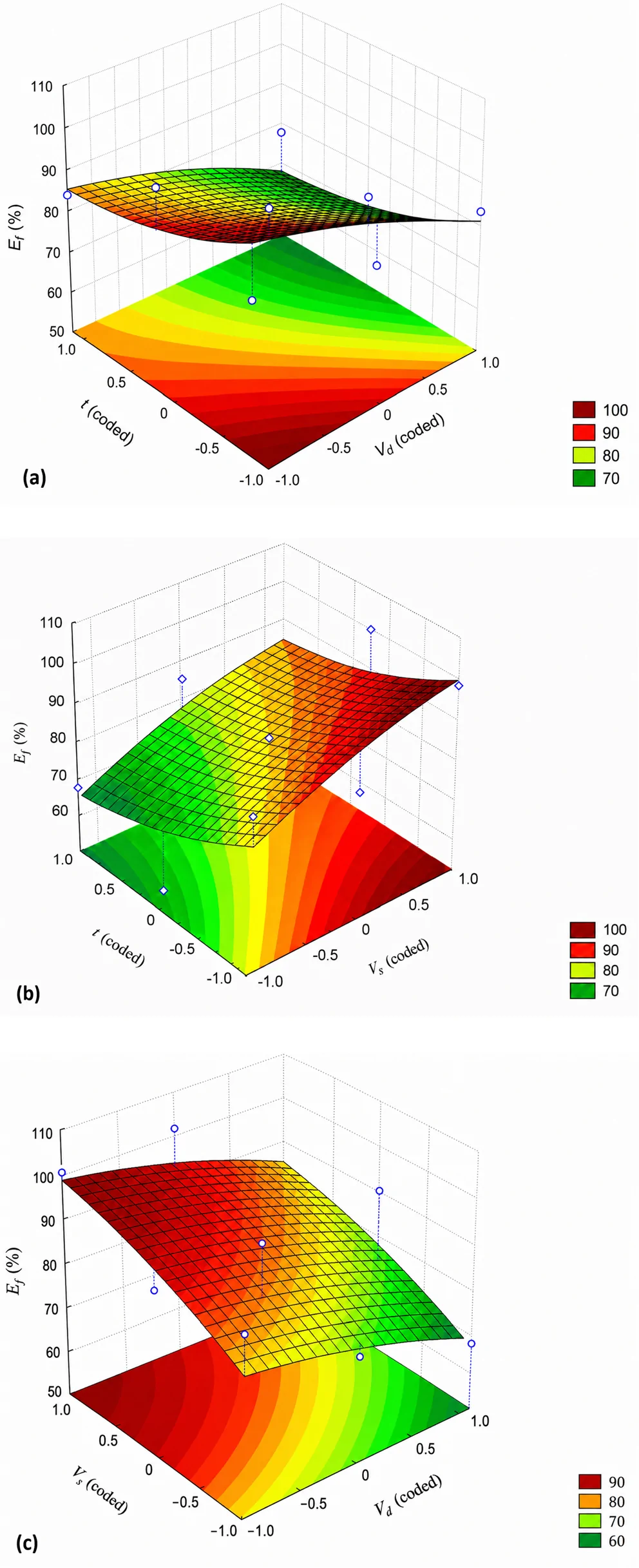
Response surface and contour plots illustrating the synergistic effects on recovery efficiency: (a) agitation time vs. surfactant volume; (b) agitation time vs. solvent volume; (c) solvent volume vs. surfactant volume. Fixed variables are kept at their central points (level 0).

In Figure 5a, the interaction between time and surfactant volume shows that the highest recovery efficiencies were obtained at the lowest values of both parameters. This behavior occurs because excessive surfactant concentrations can form a nanodemulsifying film around water droplets that inhibits coalescence and hinders phase separation.^45^ Figure 5b shows that at lower centrifugation times, the best efficiencies were achieved with the highest volumes of natural gas condensate studied. The positive influence of increasing solvent volume is attributed to its ability to reduce oil viscosity and weaken intermolecular interactions among heavy components such as asphaltenes and resins.^7,66^ Figure 5c further confirms that the best efficiencies were obtained when solvent volume was increased and surfactant volume was reduced. This optimized chemical balance is essential for breaking and dispersing solid aggregates formed by bound water, enabling the release of hydrocarbons that are typically resistant to conventional solvent extraction.^5,14^ As supported by recent literature on production chemistry, the integration of statistical optimization and predictive modeling is a robust approach to resolve complex interactions and improve treatment efficiency in oilfield operations. Figure 6 presents the predicted vs. observed values used to evaluate the adequacy of the selected model.^78^

**Figure 6:**
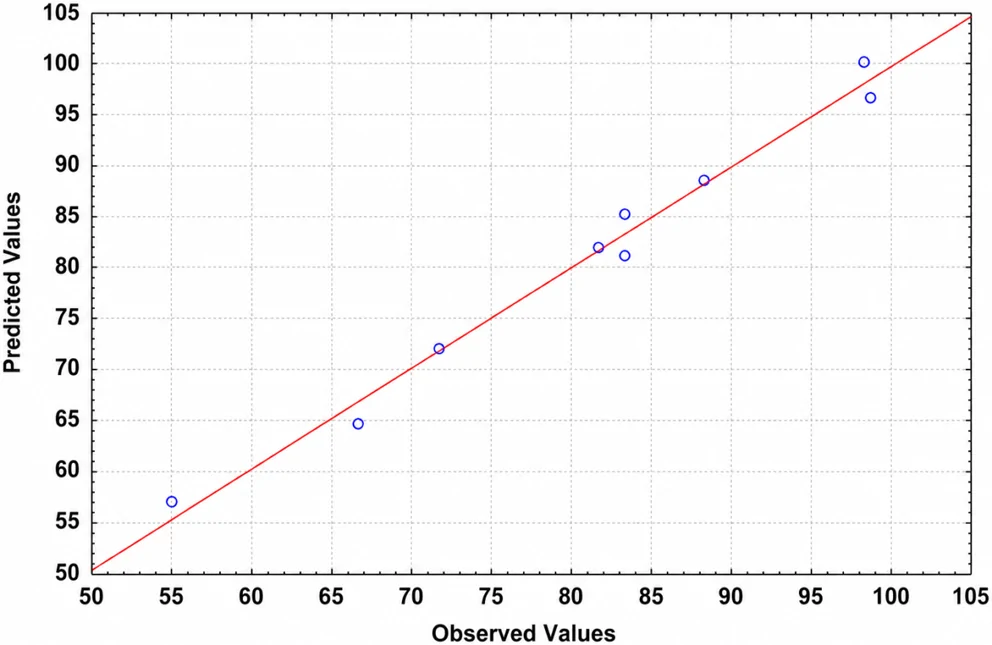
Predicted vs. observed values showing strong agreement between model estimates and experimental data.

The concentration of points near the 45° slope line indicates that the selected model does not exhibit a lack of fit and adequately represents the data. Beyond confirming the absence of systematic bias, the clustering of points along this line demonstrates that the model captures both the central tendency and the variability of the experimental responses. The absence of pronounced deviations or curvature patterns suggests that no major nonlinearities or interaction effects were left unaccounted for in the modeling process. Furthermore, the narrow dispersion of points around the reference line reinforces the model’s robustness and predictive reliability, supporting its suitability for interpreting the combined effects of solvent and surfactant volumes on recovery efficiency. This graphical behavior is fully consistent with the high *R* ^2^ value obtained and corroborates the conclusions from the ANOVA test, which indicated that the model is statistically significant and that the lack-of-fit component is not relevant. Together, these results strengthen the evidence that the chosen model provides an accurate and reliable representation of the experimental system.

## 4 Conclusions

The extraction process using natural gas condensate (C5+) combined with a surfactant in a mixer–settler system demonstrated strong technical potential for recovering hydrocarbons from residual oily sludge. Maximum efficiencies of 99.9 percent for the centrifugal route and 99.8 percent for the gravitational route confirm the robustness of the proposed methodology. This approach also enables the valorization of a natural gas processing byproduct that is often underutilized, transforming it into an efficient and low-cost extraction agent. A key contribution of this study is the first integrated demonstration that C5^+^ can function as the primary solvent in a mixer–settler system under statistically optimized conditions, replacing conventional petrochemical solvents while maintaining near-complete hydrocarbon recovery.

The study showed that optimizing operational variables is essential for achieving a high recovery performance. The best results were obtained when the maximum solvent volumes were paired with minimum surfactant dosages. Strategically reducing the surfactant concentration prevented the reverse effect of emulsion stabilization by avoiding the formation of nanodemulsifying films that inhibit droplet coalescence. The high efficiency achieved in the centrifugal route highlights the feasibility of rapid separations, while the optimized gravitational route offers a simplified and low-energy alternative for facilities with limited mechanical equipment.

The use of C5^+^ in petroleum waste treatment represents an integrated solution to both environmental and operational challenges, specifically by utilizing an internal industrial byproduct to replace conventional external solvents. While this methodology mitigates contamination risks and offers direct economic benefits through hydrocarbon recovery, it currently faces limitations related to high solvent consumption and the absence of empirical data on the long-term solvent recycling. Therefore, future research should prioritize the integration of distillation-based recovery units to evaluate the stability of C5^+^ over multiple reuse cycles and minimize the solvent loss. Additionally, advanced studies of interfacial rheology and microscopic droplet evolution are recommended to fully elucidate the synergistic mechanisms between C5^+^ and the surfactant. Overall, this study consolidates a technically viable and sustainable route for waste management, providing a foundation for future engineering optimizations aimed at achieving full industrial circularity.

## Data Availability

Data will be made available on request.
